# The Adult *Aedes aegypti* Bacterial Microbiota is Robust to Infection with the Obligate Microsporidian Parasite *Edhazardia aedis*

**DOI:** 10.1111/1462-2920.70437

**Published:** 2026-10-06

**Authors:** Dom Magistrado, Sarah M. Short

**Affiliations:** ^1^ Department of Entomology, College of Food, Agricultural, and Environmental Sciences The Ohio State University Columbus Ohio USA; ^2^ Department of Microbiology and Immunology University of Maryland School of Medicine Baltimore Maryland USA

**Keywords:** host–pathogen interactions, microbiota, microsporidia, mosquito, pathogens

## Abstract

*Edhazardia aedis* is an obligate intracellular microsporidian parasite of the arthropod vector *Aedes aegypti* , which is responsible for the spread of several vertebrate pathogens of global health importance. *E. aedis* can be highly virulent to *A. aegypti* and infection has severely detrimental effects on multiple *A. aegypti* life history traits, including longevity, body size, propensity to host‐seek and blood‐feed, and reproductive capacity. Herein, we queried the effect of *E. aedis* infection on the bacterial microbiota of adult female *A. aegypti* using high‐throughput amplicon sequencing of the 16S rRNA gene. Analysis of sequencing data revealed that the bacterial microbiota community is strikingly robust to *E. aedis* infection, as we observed no significant effect on alpha or beta diversity, differential abundance of any taxa, predicted metabolic function profile, or bacterial load. The data show that *E. aedis*, despite dramatically impacting the health and fitness of the adult female mosquito, does not affect the microbiota. These results provide unique insight into tripartite relationships (or lack thereof) between hosts, pathogens, and the microbiota.

## Graphical abstract

In ‘The adult *Aedes aegypti* microbiota is robust to infection with the microsporidian parasite *Edhazardia aedis*’, Dom Magistrado and Sarah M. Short* use high‐throughput 16S amplicon sequencing to reveal that in contrast to a multitude of other bacterial, viral, and microsporidian pathogens, infection with the microsporidian pathogen *E. aedis* does not alter the microbiota of adult female *A. aegypti* , despite dramatically altering host immune system signalling and imparting severe disease and fitness costs.

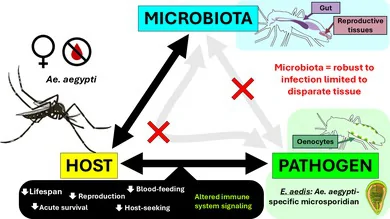

## 1 Introduction

Microbes that live in association with host organisms (i.e., the microbiota) are increasingly implicated in a holistic understanding of the biology of the host. The holobiont concept is the idea that host organisms are best understood not in isolation, but rather, as a complex or ‘meta‐organism’ comprised of both the host and the microbes associated with that host (Greer et al. 2016; Simon et al. 2019). The microbiota affect a suite of functions in hosts, playing key roles in immunity and metabolism (Belkaid and Harrison 2017; Debnath et al. 2021; Ikeda‐Ohtsubo et al. 2018), and perturbation of the microbiota (dysbiosis) typically causes disease in hosts (Petersen and Round 2014; Ram and Dastager 2019; Rosenfeld 2017). Interest in understanding the mosquito microbiota has emerged in recent years (Flores et al. 2022; Guégan et al. 2018; Minard et al. 2013; Singh and Das 2020; Zheng et al. 2023) because microbes can be exploited to suppress the spread of mosquito‐borne diseases such as dengue, Zika, chikungunya, and yellow fever. Indeed, microbes such as the endosymbiotic bacteria *Wolbachia* and the entomopathogen *Bacillus thuringiensis israelensis* have been utilized to suppress vector populations with some success (Crawford et al. 2020; Patyka and Patyka 2020). Despite these successes, mosquito‐borne disease remains a growing global public health problem, as 2024 was the worst year for dengue cases recorded in history (The Lancet 2024). Clearly, the demand for new solutions remains high. Studying mosquito‐microbiota interactions provides not only potential for uncovering novel microbial agents with direct potential for biocontrol, but also contributes to an improved, holistic framework of understanding mosquito vector physiology that accounts for the increasingly realized fundamental role of microbes.

Mosquitoes associate with microbes at every life stage (Wang et al. 2011) and the microbiota play important roles in many physiological processes. For example, the microbiota are indispensable for normal larval development (Coon et al. 2014), may mediate insecticide resistance (Arévalo‐Cortés et al. 2020; Dada et al. 2018; Omoke et al. 2021; Pelloquin et al. 2021; Soltani et al. 2017), play a role in the formation of the adult peritrophic matrix (Rodgers et al. 2017), and contribute to the digestion of both blood (Wang et al. 2011; Rodgers et al. 2017; Gaio et al. 2011) and sugar (Guégan et al. 2020; Guégan et al. 2022; Salgado et al. 2024; Villegas et al. 2023). Female mosquitoes may even beneficially engineer the microbiota of their progeny by depositing microbes into the larval habitat during oviposition (Mosquera et al. 2023). While the mosquito microbiota is taxonomically diverse and includes bacteria, viruses, fungi, and protists, bacteria are by far the best studied and have disproportionately informed the current paradigm of how resident microbiota interact with hosts (Guégan et al. 2018). Importantly, all microbes within this diverse suite have the potential to interact not only with the mosquito directly, but with other members of the holobiont as well, and this concept underpins the field of microbial ecology. Indeed, exploration of tripartite interactions between mosquitoes, resident microbiota, and microbial pathogens of human importance has revealed that the mosquito's resident bacterial, viral, fungal, and microsporidian microbiota can interfere with the success of *Plasmodium* parasites and multiple arboviruses including dengue and West Nile in establishing infection in mosquitoes (Angleró‐Rodríguez et al. 2016; Apte‐Deshpande et al. 2014; Bolling et al. 2012; Bongio and Lampe 2015; Cirimotich et al. 2011; Dickson et al. 2017; Goenaga et al. 2015; Herren et al. 2020; Ramirez et al. 2014; Tchioffo et al. 2013; Valzano et al. 2016; Wang et al. 2013).

In the present study, we investigate the impact of the microsporidian parasite *Edhazardia aedis* on the bacterial microbiota in the abdomens of the yellow fever mosquito *Aedes aegypti* . Microsporidia are fungus‐like obligate intracellular parasites that are strikingly ubiquitous and diverse, infecting myriad hosts ranging from protists to mammals (Ruan et al. 2021), including hosts from all major insect orders (Becnel and Andreadis 2014). *E. aedis* was first isolated in Puerto Rico in 1930, then later re‐discovered in Thailand in 1979 (Hembree 1979; Kudo 1930; Nasci et al. 1992). The current geographical range of *E. aedis* has not been surveyed and thus remains unknown. Naïve *A. aegypti* larvae can encounter *E. aedis* in the aquatic larval habitat through ingestion of spores released from infectious larvae (i.e., horizontal infection). After *E. aedis* enters the larval digestive tract, it invades cells and aggregates in the gastric caecae (Becnel et al. 1989). *E. aedis* is virulent to *A. aegypti*, and survival outcome is affected by larval instar development stage, food availability, and the quantity of spores ingested (Agnew and Koella 1999; Zilio et al. 2023). During mosquito metamorphosis, the mosquito gut tissue is fully regenerated and the contents of the larval gut (e.g., any remaining inoculation spores) are digested and expelled shortly after emergence (Lindh et al. 2008; Romoser et al. 2000; Moll et al. 2001). In females that survive to adulthood, *E. aedis* is limited primarily to the oenocytes of the fat body (Becnel et al. 1989; Agnew and Koella 1999; Johnson et al. 1997) until bloodfeeding, an event that initiates infection of the ovaries and infection of progeny, thereby potentiating transovarial vertical transmission of the parasite (Becnel et al. 1989). *E. aedis* undergoes development and replication in the vertically infected larvae, leading to the formation of distinctive spore cysts that, upon larval death, are released into the larval habitat to permit horizontal infection of naïve larvae, thus completing the life cycle (Becnel et al. 1989). Infection with *E. aedis* alters the whole‐body transcript abundance of genes related to immunity and metabolism at all life stages in horizontally infected female mosquitoes (Desjardins et al. 2015). It also reduces defence against opportunistic bacterial infection (El‐Dougdoug et al. 2024) and begets high fitness costs to the mosquito host via reductions in longevity, body size, propensity to host‐seek, propensity to blood feed, and reproductive capacity (Nasci et al. 1992; Barnard et al. 2007; Becnel et al. 1995; Koella and Agnew 1997). In a multitude of other host–pathogen systems, infection with diverse pathogens including microsporidians has been shown to affect the arthropod microbiota (Alberoni et al. 2023; Boissière et al. 2012; Maes et al. 2016; Mousson et al. 2010; Naree et al. 2022; Panjad et al. 2021; Paris et al. 2020; Shen et al. 2022; Villegas et al. 2018; Wei et al. 2017; Zhang et al. 2022; Zouache et al. 2012), but the effect of *E. aedis* on *A. aegypti* has not yet been explored.

In the present cross‐sectional study, we query the effects of horizontal infection with *E. aedis* on the abdominal bacterial microbiota of individual adult female *A. aegypti* (non‐blood‐fed), which primarily comprises microbes colonizing the mosquito's epithelial surfaces (i.e., gut tissues) that are acquired from the mosquito's environment. We use high throughput amplicon sequencing targeting the 16S rRNA gene followed by ecological analyses suitable for the compositional nature of sequencing data (Gloor et al. 2017) to capture infection‐induced perturbations in bacterial diversity. Because sequencing counts do not reflect the absolute abundance of starting material, we also use quantitative PCR targeting the 16S rRNA gene to approximate, and query an effect of *E. aedis* infection on, bacterial load. Overall, the data indicate that the bacterial microbiota in the adult abdomen is strikingly robust to *E. aedis* infection, as no analyses revealed any effect of infection on abundance, taxonomic diversity, or predicted metabolic functional profiles of the microbial communities. These findings contribute novel information about the nature and specificity of *A. aegypti's* physiological response to *E. aedis* infection and provide an improved understanding of the mosquito holobiont.

## 2 Results

To query how horizontal infection with *E. aedis* impacts the bacterial microbiota of *A. aegypti* (abdomens), we reared three paired sets of *E. aedis*‐treated or mock‐infected larvae in parallel from egg to adulthood and extracted DNA from *n* = 7–8 individual female mosquitoes (non‐blood‐fed) per cage shortly after eclosion (Figure 1). PCR indicated that all *E. aedis*‐treated individuals that we sampled were infected (see Materials and Methods for details); thereafter, these individuals were considered *E. aedis*(+). To characterize both the abundance and the composition of the bacterial microbiota in abdomens, we quantified and sequenced a segment of the V3‐V4 hypervariable region of the bacterial 16S rRNA gene using quantitative PCR and high‐throughput sequencing.

**FIGURE 1:**
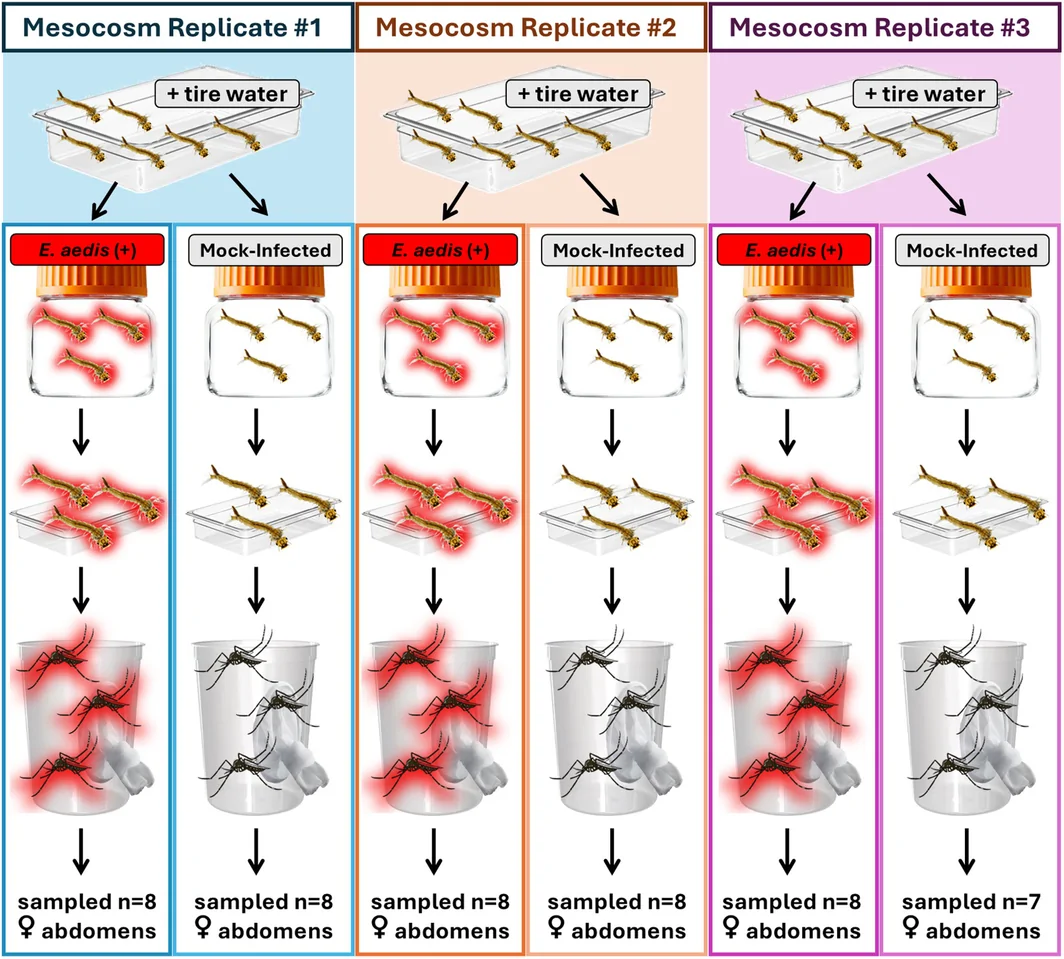
Experimental design schematic. One egg paper was used to hatch all *A. aegypti* (LVP strain) mosquitoes used in this experiment. Immediately after hatching, larvae were divided into three trays. In an effort to more closely mimic natural larval environments as opposed to standard laboratory rearing, each tray was inoculated with water collected from *Aedes* larval development sites. Larvae were reared in trays until they reached instar 2–3, then were designated to a control (mock‐infected) or *E. aedis*(+) group. Groups destined for *E. aedis* infection were horizontally infected via exposure to purified *E. aedis* spores harvested from vertically infected larvae (not shown). Following horizontal infection, larvae were transferred to standard rearing trays and monitored until pupation. Pupae were transferred to cages and allowed to eclose. At 3–5 days post‐eclosion, *n* = 7–8 abdomens from individual females were sampled from each cage and subjected to DNA extraction before sequencing.

### 2.1 Characterization of Sequencing Dataset

The final sequencing dataset used for analysis contained 5,757,955 total reads with an average of 122,510 reads per sample, and 3667 total amplicon sequence variants (ASVs) with an average of 221 ASVs per sample. Rarefaction curves generated from the final phyloseq object suggest that the samples were sequenced to a sufficient depth (Figure S1). Twenty ‘Core ASVs’ (0.01% of all ASVs) were present in all 47 samples (Table S1).

### 2.2 Taxonomic Composition

The bacterial microbiota in abdomens was dominated primarily by bacteria from the phyla Pseudomonadata and Bacillota, with a substantial proportion of reads also coming from the phylum Actinomycetota (Figure 2). Within Pseudomonadata, Gammaproteobacteria were more abundant than Alphaproteobacteria. Bacilli were the dominant class within Bacillota, and Actinobacteria was the dominant class within Actinomycetota.

**FIGURE 2:**
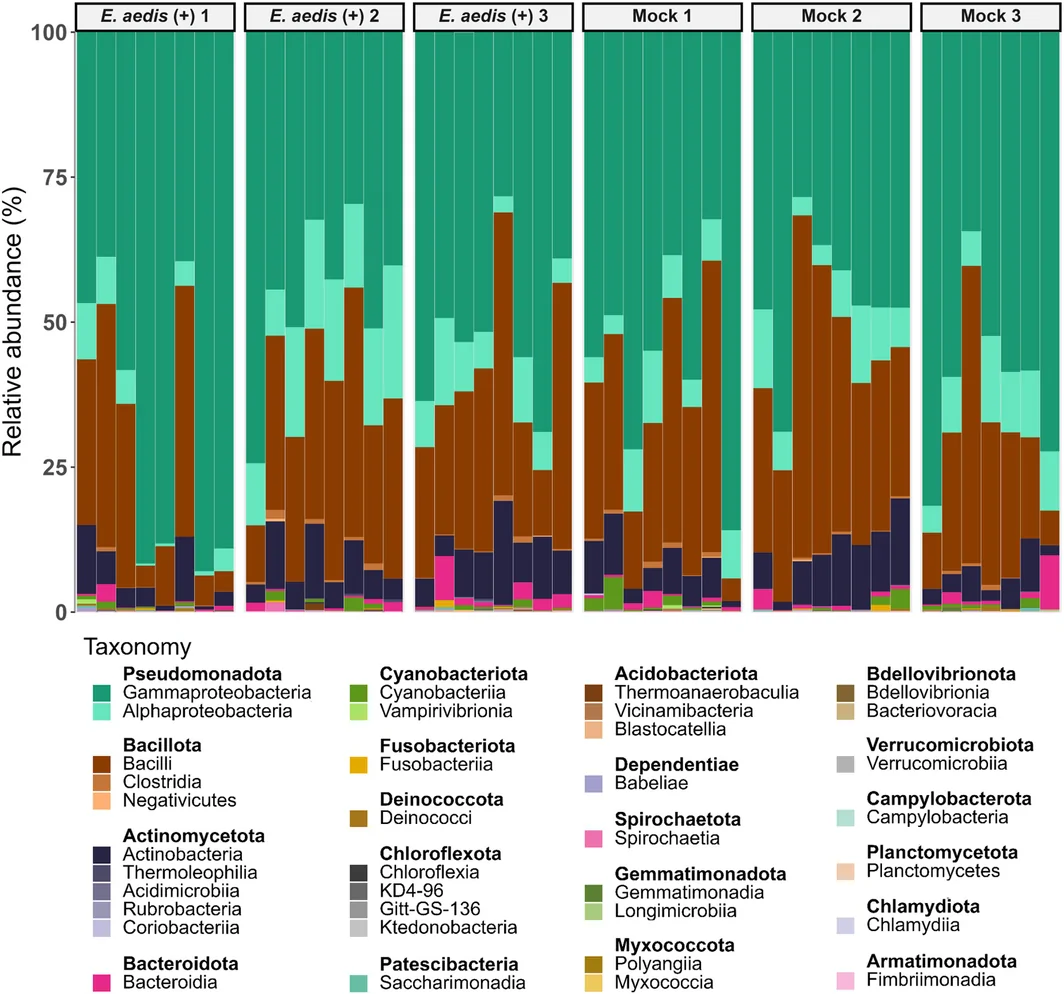
The bacterial microbiota is dominated by Pseudomonadata, Bacillota, and Actinomycetota. Individual bars represent individual samples of *n* = 1 female *A. aegypti* abdomen. The stacked bar segments and taxa labels are arranged by abundance.

Sequencing of the tire water that was used to inoculate all larval trays revealed that at the phylum level, the tire water bacterial community comprised mostly Pseudomonadata, with smaller proportions of Bacteriodota, Actinomycetota, and Planctomycetota (Figure 3C). Out of the 286 ASVs present in the tire water, 132 ASVs were also detected in mosquito samples (Figure 3A,C), while 163 ASVs were unique to the tire water inoculum (Figure 3A,C). Shared ASVs comprised approximately 50.0% of the tire water reads (90,950 out of 182,007 reads) and 57.1% (3,288,839 out of 5,757,955 reads) of the mosquito sample reads (Figure 3B). Shared ASVs constitute a majority (16 out of 20) of the Core ASVs (Table S1). At the phylum level, the composition of the Shared ASVs is similar to that of the ASVs in mosquito samples only, comprising mostly Pseudomonadata and Bacillota, with a smaller proportion of Actinomycetota (Figure 3D). ASVs unique to mosquitoes are expected to have originated primarily from the insectary environment or from maternal deposits during oviposition.

**FIGURE 3:**
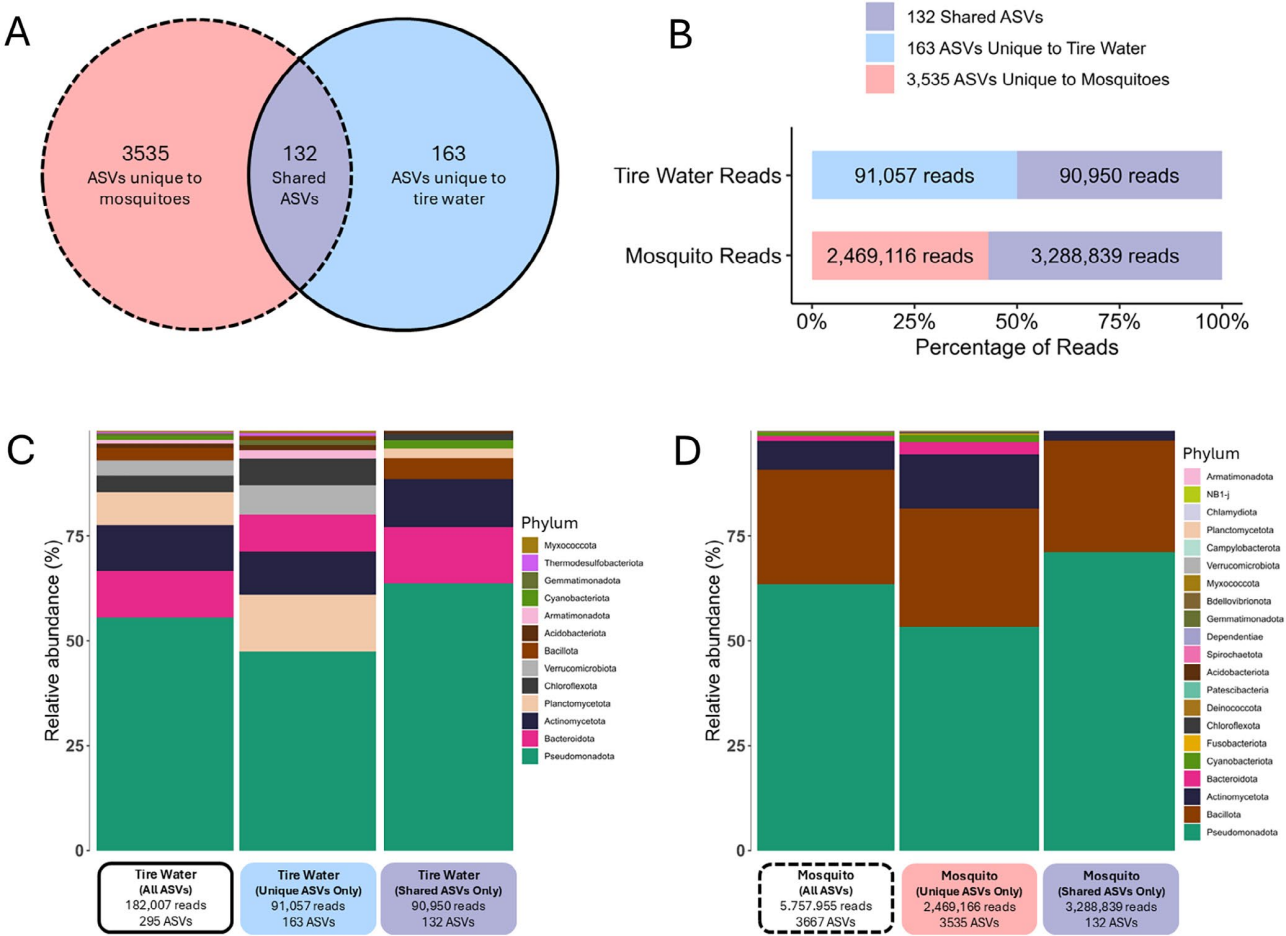
Analysis of ASVs in tire water. ASVs were (A) categorized as unique to mosquito samples, unique to tire water inoculum, or shared between the two sample types, and (B) the weights of each categorization were characterized using the number or proportion of reads assigned to each categorization within tire water or mosquito reads. Phylum‐level taxonomy is displayed for tire water (C) or mosquito samples (D) as uncategorized (left bar plots) or categorized into unique or shared ASVs (middle and right bar plots, respectively).

### 2.3 Infection With Edhazardia Aedis Does Not Alter the Abundance, Taxonomic Diversity, or Predicted Metabolic Functional Profiles of the Bacterial Microbiota in Abdomens of Female Non‐Blood‐Fed *A. aegypti*

We characterized the alpha diversity of the bacterial microbiota in each sample using Chao1, Shannon, and Simpson indices and queried the effects of *E. aedis* infection and larval mesocosm replicate on each alpha diversity index using the Wilcoxon Rank‐Sum test (for *E. aedis* infection status) or the Kruskal‐Wallis test (for larval mesocosm replicate). Neither *E. aedis* infection status nor mesocosm had a significant effect on any measure of alpha diversity of the adult bacterial microbiota (Figure 4A–C; Supporting information: File S1: Wilcoxon rank‐sum test: *p*_Chao1, *E. aedis*_ = 0.246, *p*_Shannon, *E. aedis*_ = 0.907, *p*_Simpson, *E. aedis*_ = 0.742; Supporting information: File S1: Kruskal–Wallis test: *p*_Chao1, Mesocosm_ = 0.634, *p*_Shannon, Mesocosm_ = 0.161, *p*_Simpson, Mesocosm_ = 0.126).

**FIGURE 4:**
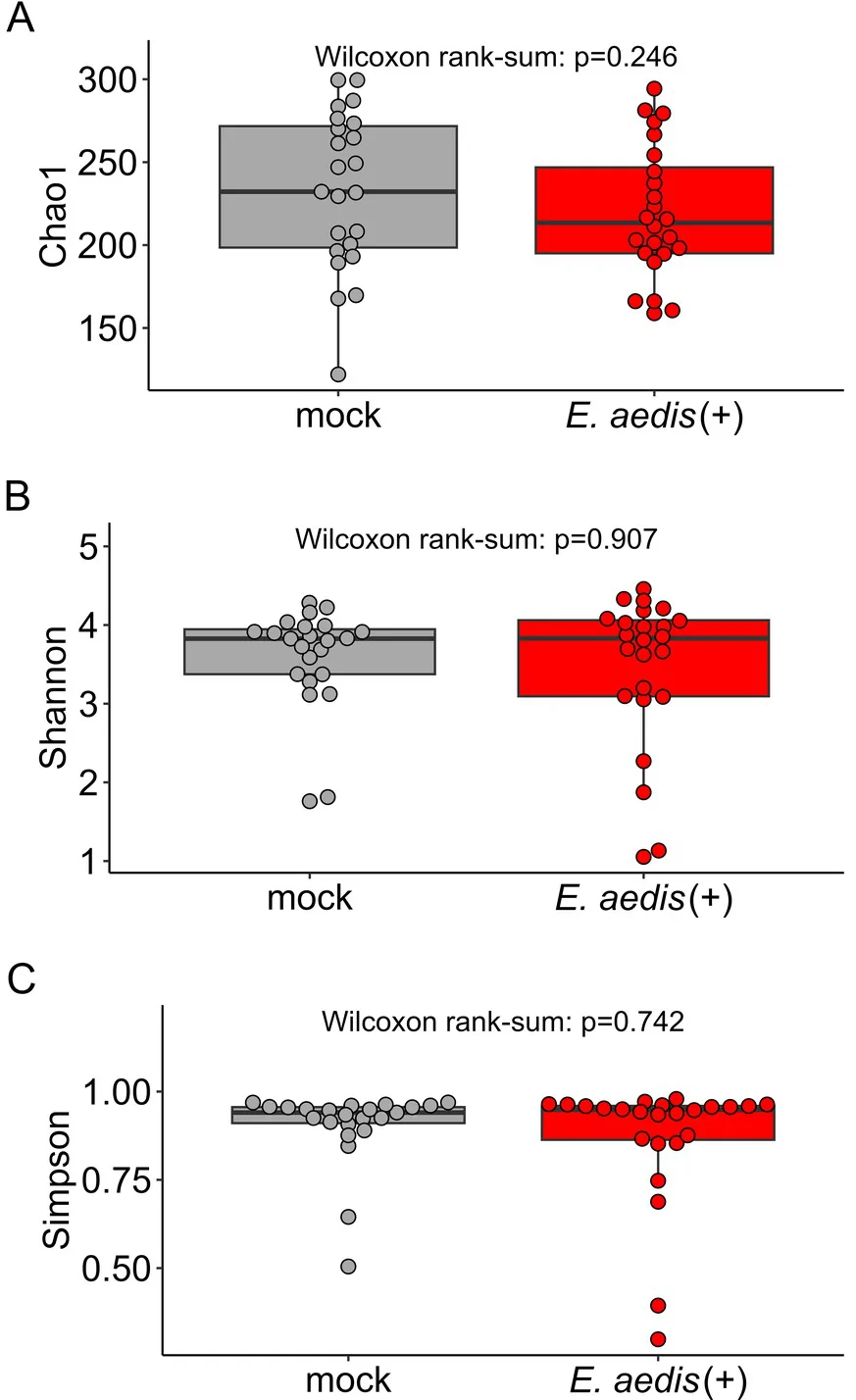
Infection with *E. aedis* does not alter the alpha diversity of the bacterial microbiota in the abdomens of *A. aegypti*. *p* values indicate the results of Wilcoxon rank‐sum statistical tests for significant differences in (A) Chao1 Index, (B) Shannon Index, or (C) Simpson Index between mock‐infected and *E. aedis*(+) samples. No differences were found at any taxonomic level; ASV level is shown in the figure.

We constructed beta diversity ordinations using a suite of distances or transformed abundance matrices: Bray Curtis distances (phylogeny‐unaware), robust CLR transformed abundance matrix (phylogeny‐unaware, appropriate for compositional data), CLR transformed abundance matrix with imputed zeroes (phylogeny‐unaware and appropriate for compositional data), and balances derived from a phylogenetic isometric log‐ratio transformation (phylogeny‐aware, appropriate for compositional data) (Figure 5A–D). To query effects of *E. aedis* infection on beta diversity of the bacterial microbiota, we used PERMANOVAs to test for significant differences between *E. aedis*(+) and mock‐infected groups, with restricted permutations to account for any effect explained by larval mesocosm replicate. PERMANOVAs revealed no significant effect of infection on beta diversity of the bacterial microbiota for any distance metric (Figure 5A–D; Supporting information: File S1: PERMANOVAs: *p*_Bray‐Curtis,*E. aedis*_ = 0.368, *p*_RCLR, *E. aedis*_ = 0.698, *p*_Zero‐imputed CLR, *E. aedis*_ = 0.245, *p*_PHILR, *E. aedis*_ = 0.428).

**FIGURE 5:**
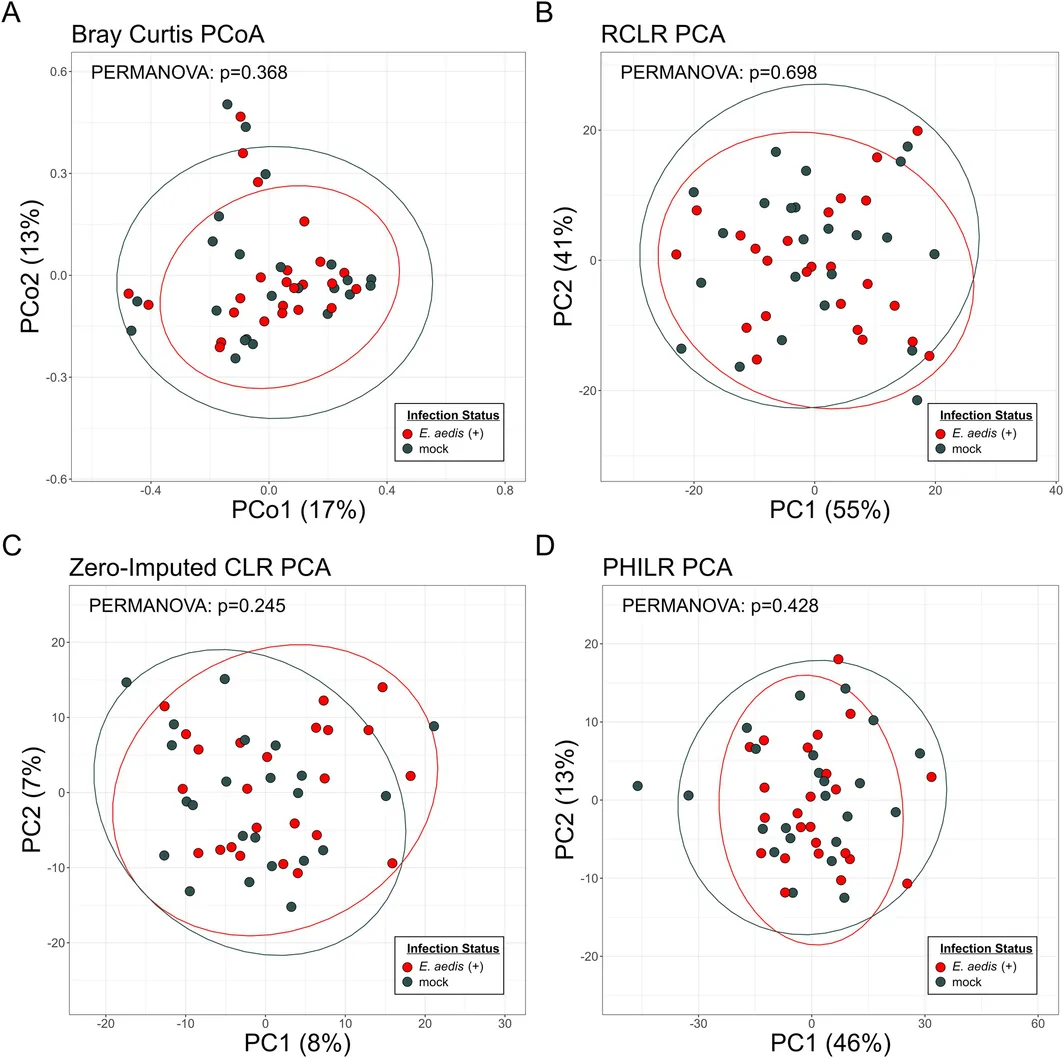
Infection with *E. aedis* does not alter the beta diversity of the bacterial microbiota in the abdomens of *A. aegypti*. Ordination analyses use (A) Bray‐Curtis distances, (B) Robust CLR distances, (C) zero‐imputed CLR distances, or (D) PHILR distances (phylogeny‐aware). *p* values indicate the results of PERMANOVA tests for significant differences between mock‐infected and *E. aedis*(+) samples. No differences were found at any taxonomic level; ASV level is shown in the figure.

We also sought to understand the effect of *E. aedis* infection on bacterial load. Because sequencing data cannot reflect the absolute abundance of starting material, we performed quantitative PCR targeting the 16S rRNA gene to estimate the bacterial load associated with each sample. Mixed effects linear regression analysis of qPCR data revealed no effect of *E. aedis* infection on bacterial load (Figure 6; Supporting information: File S1: *p*_*E. aedis*_ = 0.819).

**FIGURE 6:**
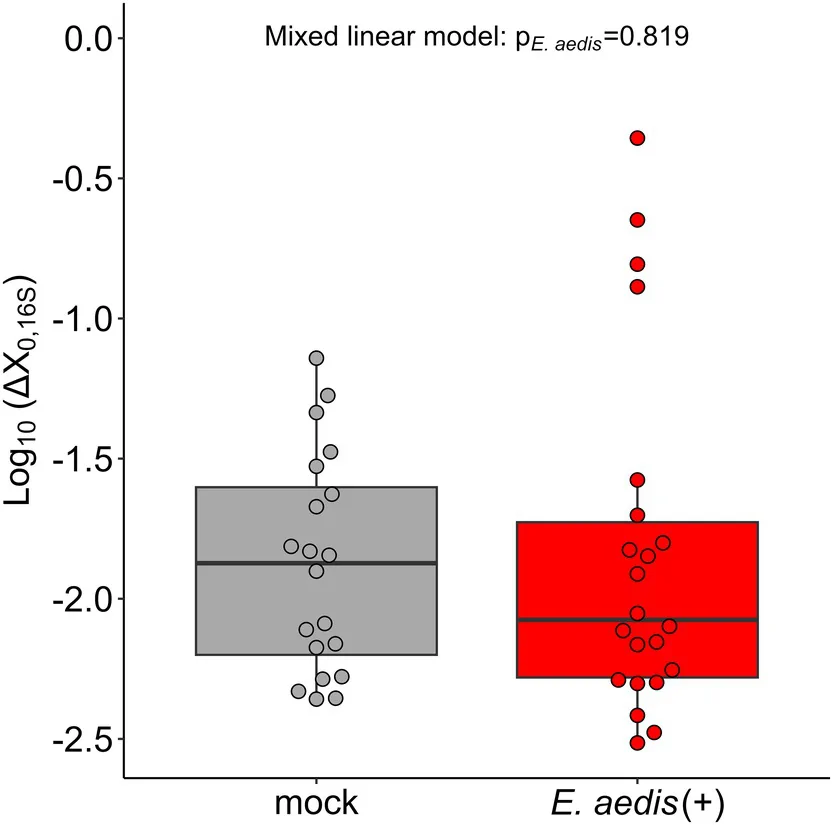
Infection with *E. aedis* does not alter bacterial load in the abdomens of *A. aegypti*. The *y*‐axis displays log10‐transformed ∆*X*_0,16S_ values, which were obtained from analysis of quantitative PCR data, and represent the starting amount of 16S bacterial DNA in each sample normalized to the amount of *RpS7* reference mosquito DNA. ∆*X*_0,16S_ values serve as a proxy for measurement of bacterial load. The *p* value indicates the result of a mixed linear model testing the effect of *E. aedis* infection on log10‐transformed ∆*X*_0,16S_ values.

We used three differential abundance analyses to determine differential abundance of taxa between Infection Status groups: ANCOM‐BC2, ALDeX2, and LinDA. Overall, differential abundance analysis did not reliably identify any taxa that were consistently differentially abundant in infected mosquitoes compared to mock‐infected. Finally, functional prediction analyses FAPROTAX and Tax4Fun2 revealed no effect of infection on the metabolic functional profiles of the bacterial microbiota.

## 3 Discussion

Our data show that the non‐blood‐fed female *A. aegypti* abdominal bacterial microbiota is robust to horizontal infection with the microsporidian parasite *E. aedis*. In contrast, a diverse set of studies conducted in *Aedes* and *Anopheles* mosquitoes show that infection with arboviruses, malaria parasites, or entomopathogenic fungi, perturb the mosquito's resident microbial community structure in various ways (Villegas et al. 2018; Zouache et al. 2012; Boissière et al. 2012; Mousson et al. 2010; Wei et al. 2017). Similar effects have also been observed in a diversity of microsporidian‐arthropod associations, including honey bees, silkworms, and Pacific white shrimp (Alberoni et al. 2023; Maes et al. 2016; Naree et al. 2022; Panjad et al. 2021; Paris et al. 2020; Shen et al. 2022; Zhang et al. 2022). A long‐standing topic of interest within this context is the mechanism by which such perturbations occur (Zouache et al. 2012). Specifically, perturbation of the microbiota may result from direct microbial interactions between specific taxa or occur secondarily to an immune response mounted against the invading microbe (Kamada et al. 2013). Our study adds novel context to this topic: unlike most of the pathogens referenced in studies above, *E. aedis* never invades gut cells in adult mosquitoes (Becnel et al. 1989; Agnew and Koella 1999; Johnson et al. 1997) and, as a result, is no longer directly interfacing with the gut tissues or gut‐associated microbiota. This is in contrast to the pathogens mentioned above, all of which interact with the gut lumen at some point in time. Interestingly, the adult mosquito does mount a marked transcriptional response to *E. aedis* infection that includes several immune signalling genes (Desjardins et al. 2015), although tissue specificity of this response (i.e., whether the response occurs in the gut) has not yet been studied. *E. aedis* infection therefore provides a unique natural pathogenic infection system that allows for the analysis of indirect microbe‐pathogen interactions occurring only as a result of effects of pathogenic infection on the host.

We previously showed that female, non‐blood‐fed *E. aedis*‐positive *A. aegypti* orally exposed to *Serratia marcescens* displayed higher *S. marcescens* loads compared to *E. aedis*‐negative mosquitoes (El‐Dougdoug et al. 2024). Notably, *S. marcescens* , which is a common gut microbiota member and opportunistic pathogen, was not detected in the present study. Taken together with the findings in the present study, the data indicate that *E. aedis* can modulate the relationship between the host and gut microbes, but the effect is context‐dependent. Two hypotheses emerge: (1) It is possible that *E. aedis* alters the mosquito response in a way that differentially affects *S. marcescens* in comparison to other microbiota members, thereby permitting *S. marcescens* to proliferate opportunistically. Under such a scenario, the results of the present study may have differed had *S. marcescens* been present in the microbiota. (2) Another explanation is that *E. aedis* alters the mosquito's induced physiological response resulting from acute bacterial exposure (e.g., oral delivery of *S. marcescens* ), but not the host mechanisms underlying the maintenance of a diverse resident microbiota community.

Importantly, more work distinguishing the mechanisms by which the mosquito host responds to invading microbes versus those by which the mosquito host shapes the resident gut microbiota is needed to integrate these findings. It is well‐established that the diversity of the microbiota is strongly influenced by both exogenous conditions (e.g., nutrition and habitat) and endogenous host factors (e.g., taxonomy, sex, and development stage), but only a limited number of studies have explored the mechanistic underpinnings of this governance (Hixson et al. 2024). More broadly, an emerging question in biology is how and why hosts may (or may not) shape the microbiota in response to stimuli such as infection, immune challenge, or disease (Byndloss et al. 2018; Litvak et al. 2018; McLoughlin et al. 2016; Schluter and Foster 2012; Wilde et al. 2024). Some evidence in the literature indicates that processes outside of the gut can influence the composition of the gut microbiota. For example, wing necrosis in *Drosophila melanogaster* leads to the overgrowth of a gut commensal, although the signalling mechanism remains unclear (Kosakamoto et al. 2020). In the social ant species *Temnothorax rugatulus*, presenting a cuticular immune challenge (lipopolysaccharide and peptidoglycan) leads to immune system signalling‐mediated decreases in gut microbiota diversity and overgrowth of bacteria from the genera *Entomoplasma* and *Pseudomonas* (Negroni et al. 2020). These studies indicate that processes shaping the microbiota are not limited to the tissues colonized by the microbiota in some arthropods. In more complex and well‐studied taxa, the opportunistically pathogenic nature of the microbiota is increasingly realized, and there are many instances of both infectious and non‐infectious disease resulting in dysbiosis (Buttó and Haller 2016; Winter and Bäumler 2023). Opportunistically pathogenic microbiota members can exploit pre‐existing host disease to establish opportunistic infections (Dey and Ray Chaudhuri 2023). *E. aedis* is highly virulent to *A. aegypti*, and substantial work shows that infection is associated with disease (Nasci et al. 1992; Barnard et al. 2007; Becnel et al. 1995; Koella and Agnew 1997). However, our data show that the microbiota do not exploit the mosquito's disease state and rather, remain unchanged. Possibly, *E. aedis* prevents the detection of a disease state in the host by gut residents. Such a strategy would benefit *E. aedis* by preventing other microbes from, for example, killing the host before *E. aedis* can complete its life cycle, further activating the immune response in a manner that is deleterious to *E. aedis*, or competing with *E. aedis* for host‐provisioned resources.

In a recent study, the presence of microsporidia in field‐caught mosquitoes in Poland was positively correlated with the presence of two bacteria belonging to *Spiroplasmataceae* and *Leuconostocaceae* in some, but not all mosquito species represented in the sample (Trzebny et al. 2023). Furthermore, microsporidia infection was associated with a significantly greater proportion of predicted metabolic function dedicated to biosynthesis of ansamycins, the biosynthesis of vancomycin group antibiotics, and the pentose phosphate pathway (Trzebny et al. 2023). Neither *A. aegypti* nor *E. aedis* were represented in the study. Consistent with our study however, Trzebny et al. did not identify any effects of microsporidia infection on alpha or beta diversity of the bacterial microbiota, suggesting that some mosquito‐associated microsporidia do not cause overall microbial community shifts in hosts. The findings herein and those reported by Trzebny et al. contrast with reports of microbiota perturbations caused by microsporidia infection in non‐mosquito arthropod hosts (Alberoni et al. 2023; Maes et al. 2016; Naree et al. 2022; Panjad et al. 2021; Paris et al. 2020; Shen et al. 2022; Zhang et al. 2022). It is possible that this is a phenomenon attributable to an unknown biological feature preventing dramatic perturbation of the host‐associated microbiota that is unique to mosquito‐infecting microsporidia. Regarding the effects reported by Trzebny et al. on *Spiroplasmataceae* and *Leuconostocaceae*, it is possible that only some microbiota members are capable of proliferating opportunistically during certain microsporidian infections, and none of these bacteria were present in the microbiota associated with our mosquitoes. It also seems plausible that some microsporidian infections render the gut a more permissive environment (e.g., nutritionally) for select members of the microbiota such as *Spiroplasmataceae* and *Leuconostocaceae* that were absent from our study. In addition, while the mosquitoes used in our study were non‐blood‐fed, field‐collected mosquitoes may have blood‐fed one or multiple times. Multiple mosquito‐associated microsporidian species undergo important life cycle progression in response to blood feeding, including *E. aedis*, *Amblyospora connecticus*, and *Microsporidia MB* (Becnel and Andreadis 2014; Onchuru et al. 2024). Because the blood meal is inextricably connected to *A. aegypti* reproduction and immunity (Foster 1995; Hurd et al. 1995; Kelly et al. 2025; Magistrado et al. 2023; Santiago et al. 2017; Schwenke et al. 2016) and transiently but dramatically reshapes the diversity of the gut microbiota (Gusmão et al. 2010; Muturi et al. 2021; Oliveira et al. 2011), investigating an effect of *E. aedis* infection in blood‐fed mosquitoes (in which *E. aedis* has colonized the ovaries) would be of interest.

## 4 Conclusion

The data indicate that the adult female *A. aegypti* bacterial microbiota is unaltered in response to horizontal infection with *E. aedis*, a microsporidian pathogen that causes severe fitness defects in its host *A. aegypti*. Interestingly, the absence of an effect of infection on the indigenous microbiota in this system contrasts with a multitude of diverse arthropod‐pathogen systems in which the microbiota is altered by pathogenic infection, including microsporidian pathogens (Alberoni et al. 2023; Boissière et al. 2012; Maes et al. 2016; Mousson et al. 2010; Naree et al. 2022; Panjad et al. 2021; Paris et al. 2020; Shen et al. 2022; Villegas et al. 2018; Wei et al. 2017; Zhang et al. 2022; Zouache et al. 2012). However, our findings are consistent with a prior study in mosquitoes that also revealed no impact of microsporidia infection on mosquito bacterial community structure (Trzebny et al. 2023). Additional research is needed to uncover the mechanisms underlying host regulation of the microbiota and how such regulation may or may not intersect with infection‐induced changes in host physiology (e.g., immunity and metabolism). Overall, the present study motivates continued investigation into *E. aedis* and provides insight into tripartite interactions (or a lack thereof) between vector mosquitoes, pathogens, and the microbiota.

## 5 Materials and Methods

### 5.1 Mosquito MaintenanceLive

Throughout the experiment, all mosquitoes described in current and subsequent sections were maintained in a 27°C chamber with 80% relative humidity under a 14 h:10 h light: dark cycle.

### 5.2 *Edhazardia aedis* Spore Isolation and Purification

*E. aedis* spores were harvested from vertically infected *A. aegypti* (LVP strain) as described in Grigsby et al., 2020 (Grigsby et al. 2020). To obtain spores for horizontal infection, eggs laid by *E. aedis*‐infected females were hatched in reverse osmosis (RO) water in a vacuum chamber. Upon hatching, larvae were thinned into 12.75″ × 10.44″ × 2.5″ polycarbonate trays (Cambro, Huntington Beach, CA) containing RO water at a density of 100 individuals per tray. At this time, each tray was given a pinch of fish food (Tetramin) and half of a green cat pellet (9 Lives). Larvae were food restricted, but not starved, only for the purpose of extending time to pupation and permitting enough time for the development of uninucleate *E. aedis* spores (Grigsby et al. 2020).

At day 10 post‐hatch, 10 larvae were collected, washed with DI (deionized) water, and homogenized. Each individual homogenate was visually examined under a microscope and evaluated for the presence of uninucleate spores. The presence of spores was confirmed in 7 out of 10 of the larvae. At this time, 50 larvae were collected, washed with DI water, and homogenized in 1 mL of DI water using a glass pestle. The presence of uninucleate spores in the homogenate was confirmed. Spores were then purified via Ludox density gradient assay as described in Solter et al. (2012). Briefly, the gradient was formed by layering 500 μL DI water on top of 500 μL Ludox reagent in a 1.5 mL microcentrifuge tube, laying the tube onto its side for 2 h to permit gradient formation, then carefully returning the tube to an upright position to avoid disturbing the gradient. 300 μL of homogenate containing the spores was then layered on top of the gradient in the tube and centrifuged at 16,000×*g* for 30 min. The resulting white band containing spores was harvested from the tube column under a flame and transferred to a 1.5 mL Eppendorf tube. This product was then washed twice (i.e., suspended in DI water, centrifuged at 1500×*g* for 10 min, and resuspended in fresh DI water after removal of supernatant)twice. Washing took place under a flame to avoid introducing environmental microbes into the spore solution.

Following spore purification via Ludox density gradient assay, spore density was calculated using an inverted microscope and a haemocytometer and used for horizontal infections. The spore density of the final spore solution was 240,000 spores/mL. This spore solution was used to horizontally infect experimental larvae as described below. In addition, the spore solution was serially diluted ten‐fold six times in sterile DI water. 100 μL of DNA was extracted from this dilution series using the ZymoBIOMICS DNA Miniprep Kit (Cat. #: D4300) following the manufacturer's instructions and subjected to PCR using *E. aedis*‐specific primers FQEA187 and RQEA310 (Duncan et al. 2012) (Table S2). Each 25 μL reaction contained 10.5 μL molecular grade water, 0.5 μL of each primer (10 μM concentration), 1 μL of undiluted gDNA template, and 12.5 μL DreamTaq PCR master mix (Thermo Scientific, Cat. #: FERK1081) and was performed on a T100 Thermal Cycler (Bio‐Rad Cat. #: 1861096) under the following thermocycling conditions: (1) 95°C, 3 min, (2) 35 cycles of 95°C, 30 s; 55°C, 30 s; 72°C, 30 s, and (3) 72°C, 10 min. The PCR product was run on a 1% agarose gel and the presence of target size (123 bp) amplicons throughout the dilution series was used to confirm our ability to detect the presence of as few as 24 spores (Figure S2).

### 5.3 Horizontal *Edhazardia aedis* Infections & Tissue Collection

To query the effect of horizontal infection on the abdominal bacterial microbiota of non‐blood‐fed female *A. aegypti* (LVP strain), we used a paired mesocosm replication structure in which three paired sets of *E. aedis* (+)/control (mock‐infected) larvae were reared in parallel from egg to adulthood (Figure 1). Horizontal infection of experimental mosquito larvae with *E. aedis* was performed as described in (Grigsby et al. 2020). To prepare experimental mosquitoes, an unbleached egg paper was hatched in RO water placed in a vacuum chamber. Upon hatching, larvae were thinned into three 12.75″ × 10.44″ × 2.5″ polycarbonate trays (Cambro, Huntington Beach, CA) containing 1 L RO water at a density of 300 individuals per tray. At this time, 0.5 mL of tire water from an *Aedes* larval development site (tire water containing *Aedes* collected in Baltimore, MD September 2016 and stored in 25% glycerol at −70°C since collection) (MacLeod et al. 2021) was added to each tray for the purpose of inoculating the laboratory aquatic larval environment with a suite of microbes found in natural *Aedes* larval environments. An aliquot of this tire water inoculum was submitted for sequencing. At this time, each tray was given a pinch of fish food (Tetramin) and green cat food pellets (9 Lives) ad libitum. At 3 days post‐hatching, larvae were randomly split and designated to an *E. aedis* infection group or a control (mock infection) group. To accomplish horizontal infection with *E. aedis*, 150 larvae were transferred to wide‐mouth 500 mL clear borosilicate glass bottles (VWR Cat. #: 75871‐304) containing 150 mL RO water and 3 mL freshly prepared liquid food slurry (liquid food slurry recipe: 1.2 g liver powder + 0.8 g brewer's yeast powder + 100 mL DI water). Infection groups received 313 μL of the 240,000 spores/mL purified spore solution, which delivered approximately 75,000 spores to each bottle. Mock infection groups received the same treatment without the addition of *E. aedis* spores. Larvae were maintained in bottles for 24 h. Following spore exposure in bottles, bottle contents were then transferred to 12.75″ × 10.44″ × 2.5″ polycarbonate trays (Cambro, Huntington Beach, CA) and supplemented with 900 mL RO water. Larvae were then reared with ad libitum access to green cat food pellets (9 Lives). Trays were checked daily to monitor pupation. Individuals that pupated between 6 and 8 days post‐hatch were removed from trays daily with sterile transfer pipettes, washed with RO water, and transferred to sterile cups containing RO water and allowed to eclose. Adults were housed in 88 oz. polypropylene container cages and provided with filter‐sterilized 10% sucrose. The pupal water was replaced with fresh RO water daily until cups were removed.

Tissues were collected in a single instance when adults were aged 3–5 days post‐eclosion. From each cage, *n* = 7–8 individual females were aspirated and cold‐anaesthetized. Individuals were externally sterilized in a 3% bleach solution for 15 s, then rinsed twice with filter‐sterilized 1X PBS. Abdomens were removed from the individuals beneath a microscope, placed into a 1.5 mL microcentrifuge tube containing 150 μL lysis buffer from the ZymoBIOMICS DNA Miniprep Kit (Cat. #: D4300), homogenized, and stored at −80°C before DNA extraction per the manufacturer's protocol. Dissection tools were cleaned with 70% ethanol and Kimwipes in between dissections.

We validated the infection status of *E. aedis* (+) females by subjecting all genomic mosquito DNA samples to PCR and gel electrophoresis using *E. aedis*‐specific primers FQEA187 and RQEA310 (Duncan et al. 2012) (Table S2) as described in the previous section. The assay confirmed the presence of *E. aedis* in all individuals exposed to infection treatment (Figure S3). The assay also indicated the absence of infection in the control individuals; however, a faint signal is present in four out of the eight control individual samples in replicate 3 (Figure S3). It is possible that DNA contamination or off‐target DNA amplification led to this signal. However, given that the exclusion of these samples does not alter the findings, we do not believe that the source of this signal interfered with the validity of the dataset or biological findings presented. One limitation of the study is the absence of a secondary method of infection confirmation. Future studies should include, for example, histological examination of tissues to confirm the presence of the parasite.

### 5.4 DNA Extraction, Library Preparation, and Sequencing Data

Total DNA was extracted from mosquito abdomens, ‘blank’ samples, and the tire water inoculum using the ZymoBIOMICS DNA Miniprep Kit (Cat. #: D4300) following the manufacturer's instructions. Following DNA extraction, amplicon PCR was performed in‐house using MyTaq Red Mix and primers 341F and 805R targeting the V3‐V4 region of the 16S rRNA gene (Klindworth et al. 2013) (Table S2). Each 50 μL reaction contained 13 μL molecular grade water, 1 μL of each primer (10 μM concentration), 10 μL of undiluted gDNA template, and 25 μL MyTaq Red Mix PCR master mix (Meridian Bioscience, Cat. #: BIO‐25043) and was performed on a T100 Thermal Cycler (Bio‐Rad Cat. #: 1861096) under the following thermocycling conditions: (1) 95°C, 3 min, (2) 37 cycles of 95°C, 30 s; 55°C, 30 s; 72°C, 30 s, and (3) 72°C, 10 min. PCR product was visualized on a 1% agarose gel and the target size amplicons were extracted using the Qiagen MinElute Gel Extraction kit (Cat. #: 28604) following the manufacturer's instructions. Amplicons were submitted to NC State Genomic Sciences Laboratory for subsequent library preparation following the Illumina 16S Metagenomic Sequencing Library Preparation guide (*16S Metagenomic Sequencing Library Preparation: Preparing 16S Ribosomal RNA Gene Amplicons for the Illumina MiSeq System* 2013). Following library preparation, samples were sequenced on an Illumina NextSeq 2000 platform (300 base pair paired‐end reads). Reads were demultiplexed at NC State Genomic Sciences Laboratory with a 1 base pair error tolerance for each index.

### 5.5 Bioinformatic Analysis: Quality Control and Data Processing

Cutadapt (Martin 2011) with regular sequence types was used to trim primer and adapter (Table S2) contamination, as well as polyGs, from reads. Any forward reads containing reverse primers/adapters in the forward orientation or forward primers/adapters in the reverse complement orientation were then removed from the dataset using a custom code in R (Supporting information: File S1). The complementary process was repeated for the reverse reads. Phred quality score bins were identified as: 0, 9, 20, and 34. Following data cleanup, the DADA2 R package (Callahan et al. 2016) was used for filtering, dereplication, sample inference, chimera identification, and merging of paired‐end reads. Briefly: (1) Reads were first filtered with filterAndTrim() with default parameters without truncation, (2) Error rates were learned using default parameters, (3) The core dada2 sample inference algorithm was applied with pseudo‐pooling, (4) reads were merged with a 20 bp overlap requirement, (5) an amplicon sequence variant (ASV) table was constructed, (6) chimeras were removed, and (7) taxonomy was assigned using IDTAXA (Murali et al. 2018) trained against the SILVA SSU r138 training set (Yilmaz et al. 2014) downloaded from DECIPHER Bioconductor package (Wright 2017). A phyloseq object was created from the ASV table using the phyloseq package in R. ASVs with undefined kingdom‐level classification and ASVs assigned to mitochondria or chloroplasts were removed from the dataset. Next, the decontam R package (Benjamin Callahan NMD 2018; Davis et al. 2018) was used to identify contaminants using the frequency method, which was implemented using index reaction DNA concentration values generated during sequencing library preparation. Finally, any ASVs that did not appear more than 10 times total in the dataset were removed from the phyloseq object. The resulting phyloseq object was used in all subsequent analyses. We recorded the number of reads lost at each step of quality control and data processing (Figure S4; Supporting information: File S2). Code for all bioinformatic analysis can be found in Supporting information: File S1. The final phyloseq object used in subsequent analyses can be found in Dryad (https://doi.org/10.5061/dryad.sbcc2frnf).

### 5.6 Statistical Analysis of the Sequencing Dataset

All statistical analyses were performed using R version 4.4.1 (R Core Team 2025) and R Studio version 2025.09.2 (Posit team 2025). R code for all statistical analyses can be found in Supporting information: File S1. The phyloseq object used in analyses can be found in Dryad (https://doi.org/10.5061/dryad.sbcc2frnf).

Ecological analyses were performed at multiple taxonomic levels. In all instances, merging of the dataset was accomplished using the tax_glom() function from the R package phyloseq (McMurdie and Holmes 2013).

To assess the effect(s) of *E. aedis* infection on alpha diversity of the microbiota, taxa richness was characterized using the Chao1 metric, while combined taxa richness and evenness was characterized using the Shannon and Simpson Indices. We then used Wilcoxon Rank‐Sum tests to evaluate effects of infection on each alpha diversity index. We also used a Kruskal–Wallis test to evaluate any variation in alpha diversity explained by larval mesocosm replicate.

To query the effect(s) of *E. aedis* infection on beta diversity, we constructed ordinations with four distances or transformed ASV tables: We constructed a PCoA using Bray‐Curtis distances, as this is a common approach in the field. But because sequencing data is compositional data (CoDa) (Gloor et al. 2017), we also constructed two compositional data‐appropriate phylogeny‐unaware ordinations with log‐ratio transformed ASV tables and applied two different zero‐handling approaches: (1) a robust centered log‐ratio (rclr) transformation, in which only non‐zero values are transformed, and (2) a centered log‐ratio (clr) transformation on an ASV table in which the zeroes are imputed using Bayesian‐multiplicative replacement, implemented using the cmultRepl() function from the zCompositions package (Palarea‐Albaladejo and Martín‐Fernández 2015). Finally, we used a CoDa‐appropriate method to construct a phylogeny‐aware ordination using balances derived from a phylogenetic isometric log‐ratio (PhILR) transformation (Silverman et al. 2017). We assessed the effect of *E. aedis* infection on the beta diversity of the microbiota using PERMANOVA tests, implemented via the adonis2() function from the R package vegan (Dixon 2003; Oksanen et al. 2001). We restricted permutations within each larval mesocosm replicate to account for any effects of replicate when assessing an overall effect of infection (Anderson and Braak 2003).

To query differential abundance of taxa, we used three differential abundance tools that are appropriate for compositional data: ANCOM‐BC2 (Lin et al. 2022; Lin and Peddada 2020; Lin and Peddada 2024), ALDeX2 (Gloor et al. 2016), and LinDA (Zhou et al. 2022). All differential abundance analyses were performed at all taxonomic levels. In order for a taxon to be considered differentially abundant, we required identification that the taxon be identified by more than one tool.

We performed functional prediction analyses using FAPROTAX (Louca et al. 2016) and Tax4Fun2 (Wemheuer et al. 2020).

### 5.7 Bacterial Load Estimation Using 16S qPCR

We also sought to understand the effect of *E. aedis* infection on bacterial load. Because sequencing data are compositional and cannot reflect the absolute abundance of starting material, we used quantitative PCR assays targeting the 16S rRNA bacterial gene and the *RpS7* mosquito gene (VectorBase: AAEL009496), with *RpS7* serving as the reference gene, to estimate the bacterial load in each sample. Reactions were performed on a Bio‐Rad CFX96 Touch Deep Well Real‐Time PCR Detection System in a volume of 10 μL containing 0.5 μL of each primer (the final concentration of each forward and reverse primer was 300 nM for *RpS7* and 500 nM for 16S), 4 μL of undiluted genomic DNA template, and 5 μL of PowerUp SYBR Green Master Mix for qPCR (Applied Biosystems Cat. #: A25741). The primer sequences were obtained from MacLeod et al., 2021 (MacLeod et al. 2021). Primer sequences can be found in Table S2. Cycling parameters for *RpS7* were: 2 min at 50°C + 2 min at 95°C + (15 s at 95°C + 1 min at 56°C) × 40 cycles, followed by a dissociation (melt curve) step. Cycling parameters for 16S were: 2 min at 50°C + 2 min at 95°C + (15 s at 95°C + 15 s at 58°C + 1 min at 72°C) × 40 cycles, followed by a dissociation (melt curve) step. We performed melt curve analysis and agarose gel electrophoresis on representative qPCR products for both genes to validate the presence of a single product of the expected size. For each qPCR assay designed, we calculated an *E*_Amp_ (amplification efficiency) value using standard curves collected from at least three technical replicates. For *RpS7*, *E*_Amp,*RpS7*_ = 90%, and for 16S, *E*_Amp,16S_ = 95%. Standard curve dilution series were generated over a sufficiently wide range such that sample Cqs fell within the range of Cqs in the standard curves. Melt curve analysis was performed on all sample reactions and confirmed the presence of a single peak. Two or more technical replicates were performed per sample per gene with an inclusion criterion of 0.5 Cq distance from the average. We used the *X*_0_ method (Thomsen et al. 2010) to compute values reflecting the abundance of 16S DNA standardized to the abundance of *RpS7* DNA for each sample. Due to insufficient remaining genomic DNA template, we could not obtain qPCR data for 5 out of the 47 samples (three control samples and two *E. aedis*(+) samples), so these samples were excluded from analysis. Prior to analysis of ΔX_0,16S_, we confirmed that *E. aedis* treatment had no effect on the abundance of the reference gene *RpS7*. To assess the effect of *E. aedis* infection on bacterial load, we applied a log transformation to ΔX_0,16S_ values to improve normality, then built a mixed effects linear model using *E. aedis* infection status to predict log_10_(ΔX_0,16S_) with random intercepts for larval mesocosm replicates. The qPCR data can be found in Supporting information: File S3.

## Author Contributions

**Dom Magistrado:** conceptualization, data curation, formal analysis, funding acquisition, investigation, methodology, project administration, software, validation, visualization, writing – original draft, writing – review and editing. **Sarah M. Short:** conceptualization, data curation, formal analysis, funding acquisition, investigation, methodology, project administration, resources, supervision, validation, writing – review and editing.

## Funding

This work was supported by the National Institutes of Health National Institute of Allergy and Infectious Diseases (grant number R21AI174093 and R01AI189572), The Ohio State University Infectious Disease Institute, and The Ohio State University College of Food, Agricultural, and Environmental Sciences (Internal Grants Program grant number 2023018). This material is based upon work supported by the National Science Foundation Graduate Research Fellowship Program under grant no. DGE‐1343012. Any opinions, findings, and conclusions or recommendations expressed in this material are those of the author(s) and do not necessarily reflect the views of the National Science Foundation. The funders had no role in study design, data collection and interpretation, or the decision to submit the work for publication.

## Ethics Statement

The content and authorship of the submitted manuscript have been approved by all authors, and that all prevailing local, national and international regulations and conventions, and normal scientific ethical practices, have been respected.

## Conflicts of Interest

The authors declare no conflicts of interest.

## Supporting information


**Figure S1:** Rarefaction curves generated from the final phyloseq object.

**Figure S2:** Establishing *E. aedis* limit of detection with PCR and gel electrophoresis. 100 μL DNA was extracted from each sample in a tenfold dilution series of the final spore solution (240,000 spores/mL) used for horizontal *E. aedis* infections and subjected to PCR with *E. aedis*‐specific primers FQEA187/RQEA310 and gel electrophoresis analysis to confirm our ability to detect the presence of as few as 24 spores. 1 Kb Plus DNA Ladder was used (Invitrogen Cat. #: 10787018).

**Figure S3:** emi70437‐sup‐0003‐FigureS3.png. *E. aedis* infection confirmation in mosquito samples with PCR and gel electrophoresis. Mosquito abdomen genomic DNA was subjected to PCR with *E. aedis*‐specific primers FQEA187/RQEA310 and gel electrophoresis to confirm infection status of mosquitoes. The infection status of sample “Mock1.2” was not assessed due to insufficient remaining sample volume. 1 Kb Plus DNA Ladder was used (Invitrogen Cat. #: 10787018).

**Figure S4:** Displaying the number of reads lost at each step of bioinformatic data processing. The data are stored in a .csv file (File S2) and the R code used to generate the figure is in File S1.

**File S1:** R code used for all data processing, analysis, and visualization.

**File S2:** A .csv file storing the number of reads lost at each step of bioinformatic data processing. The data were used to generate Figure S4 using R code in File S1.

**File S3:** A .csv file storing the qPCR data.

**Table S1:** Taxonomic information for “Core” ASVs (present in all *n* = 47 mosquito samples). Asterisks denote ASVs that were also identified in tire water inoculum.

**Table S2:** Primer information. An asterisk indicates the presence of sequencing overhang adapters in the sequences, which are denoted in bold.

## Data Availability

The data that support the findings of this study are openly available in (1) the Supporting Information of this article; (2) Sequence Read Archive at https://www.ncbi.nlm.nih.gov/bioproject/PRJNA1415287, reference number PRJNA1415287; and (3) Dryad at https://doi.org/10.5061/dryad.sbcc2frnf, reference number https://doi.org/10.5061/dryad.sbcc2fr.
